# Supplementary material for: Constructing a Prognostic Model for Clear Cell Renal Cell Carcinoma Based on Glycosyltransferase Gene and Verification of Key Gene Identification
Source: Int J Mol Sci. 2025 Oct 20;26(20):10182. doi: 10.3390/ijms262010182 (PMC12563792; doi:10.3390/ijms262010182)
Supplement: Supplementary file 1 [file ijms-26-10182-s001.zip › ijms-3876272-supplementary.pdf]

A

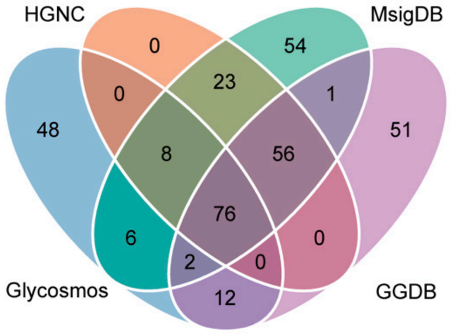

Supplement Figure S1 Functional enrichment analysis of glycosyltransferase genes. (**A**) Venn diagram showing annotated glycosyltransferase genes in GGDB, HGNC, MSigDB, and Glycosmos. Numeric values denote unique and shared genes.

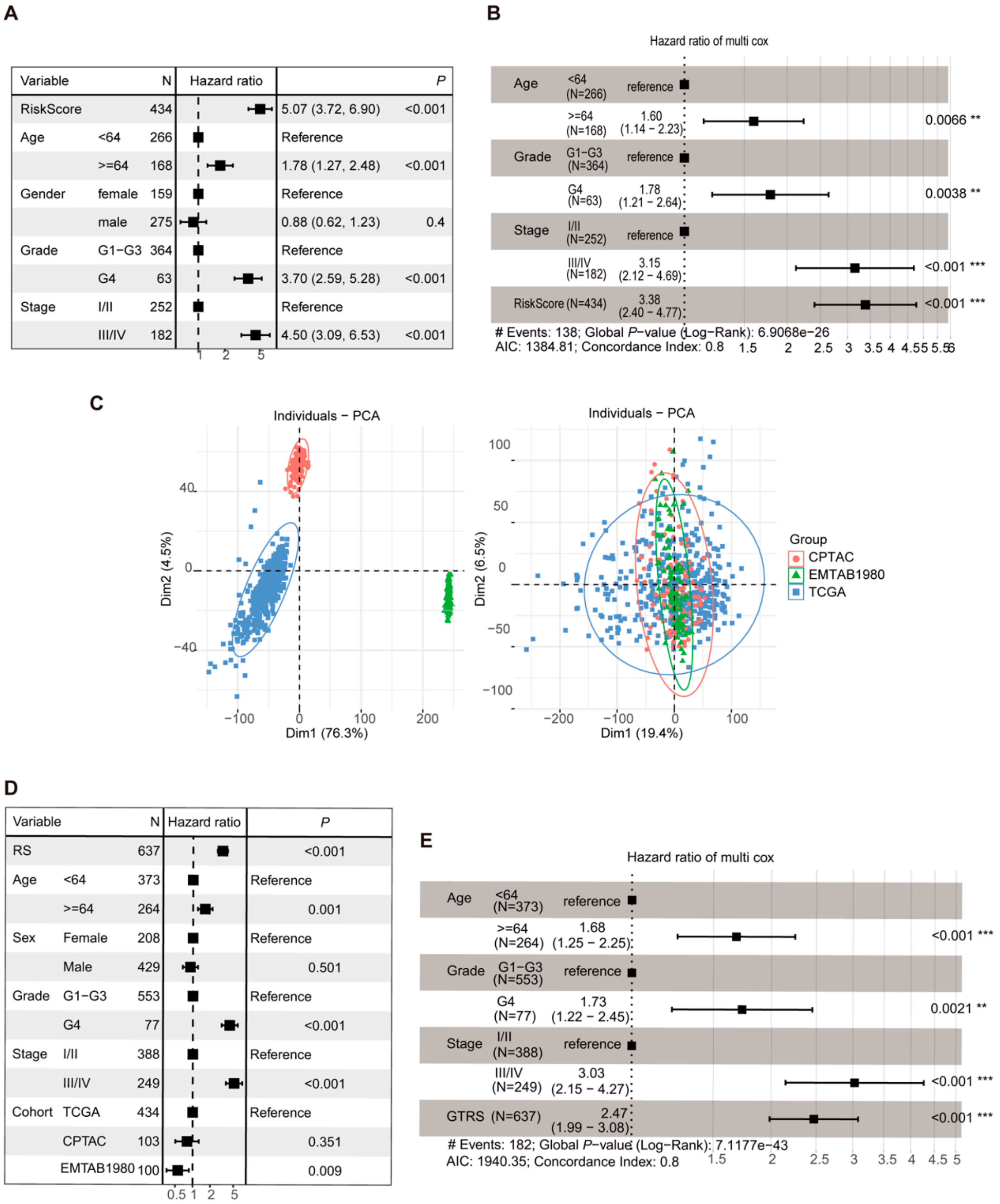

Supplement Figure S2 GTRS was an independent prognostic factor in TCGA and the pooled cohort. (**A**) Forest plot of the univariate Cox analyses for various prognostic factors. (**B**) Forest plot of the multivariate Cox analysis showing hazard ratios and 95% confidence intervals. (**C**) The PCA plots show that the data of TCGA cohort, EMTAB1980 cohort and CPTAC cohort were batch-removed and synthesized into a Meta cohort. (**D**) Univariate Cox regression analysis of the Meta cohort. (**E**) Multivariate Cox regression analysis of the Meta cohort. Hazard ratios, 95% confidence intervals, and *P* values are presented as forest plots.

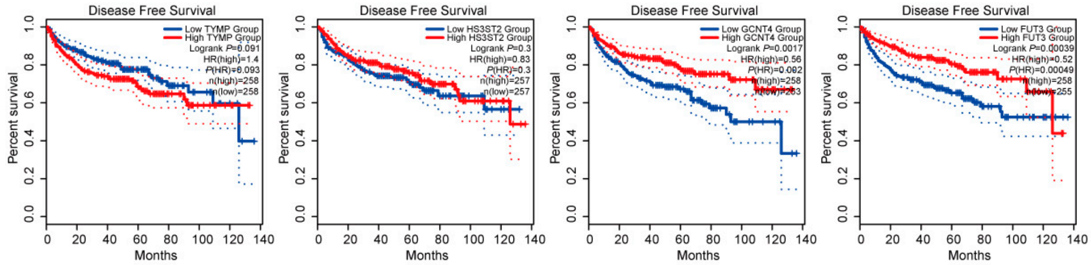

Supplement Figure S3 Kaplan-Meier curves for disease free survival in relation to the expression levels of TYMP, HS3ST2, GCNT4 and FUT3.

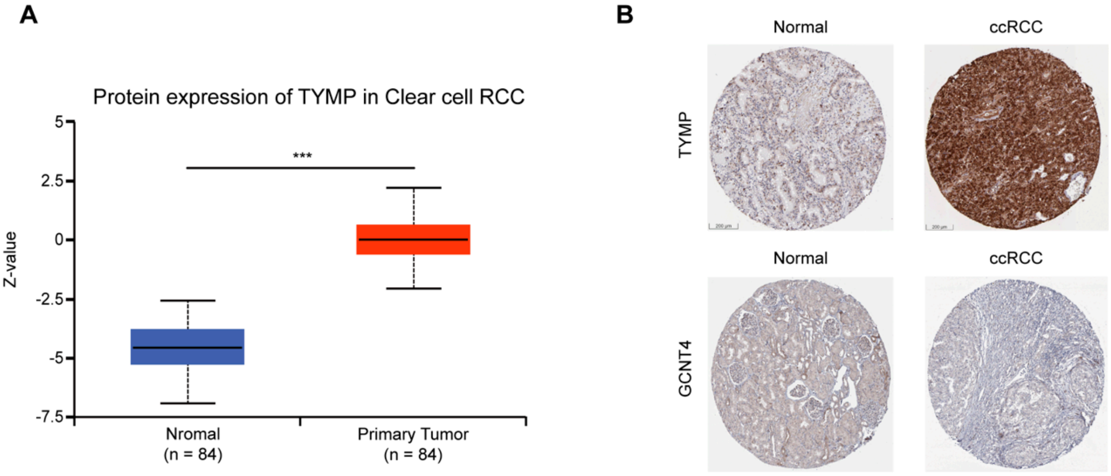

Supplement Figure S4 Protein expression of TYMP and GCNT4 in ccRCC. (**A**) The protein expression levels of TYMP in two CPTAC cohorts. (**B**) The immunohistochemical staining images of TYMP and GCNT4 protein expression.

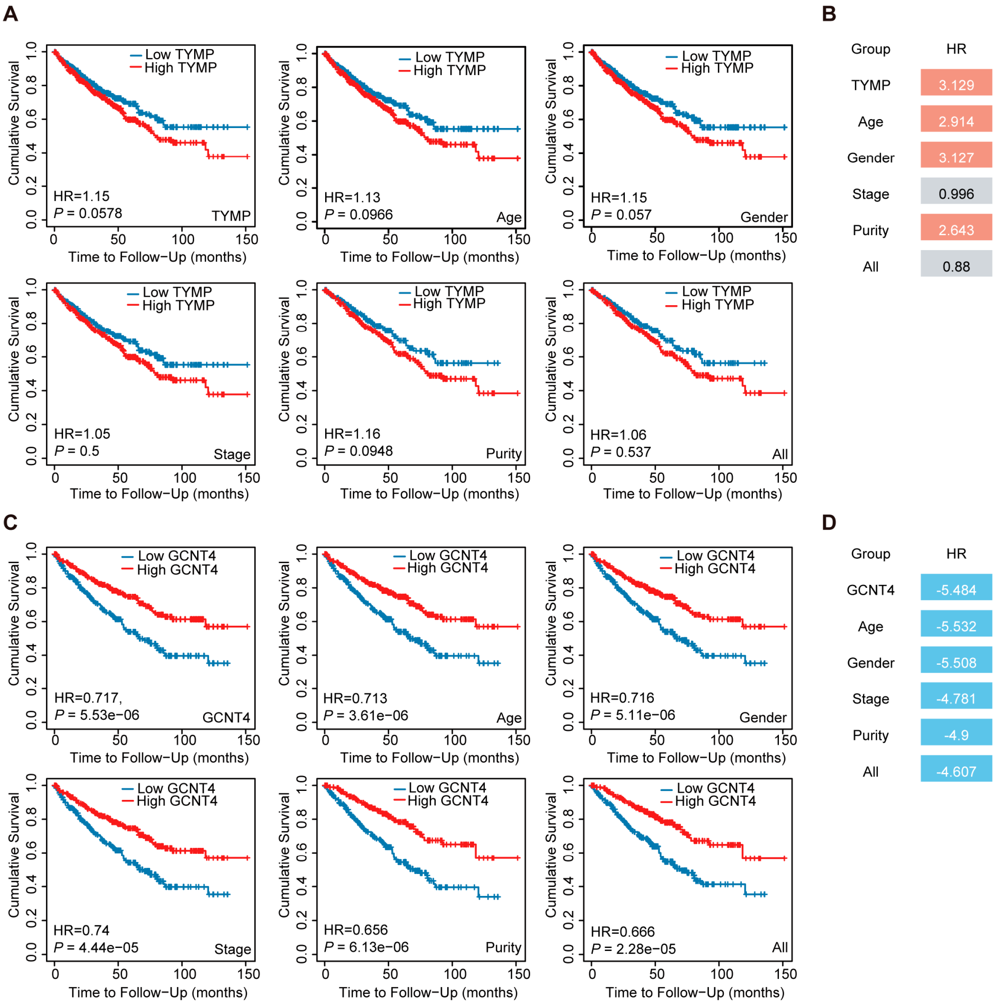

Supplement Figure S5 Association analysis of TYMP and GCNT4 gene expressions with clinical characteristics. (**A, C**) Kaplan-Meier curves (A, C) of univariate and multivariate Cox analyses for high and low expression groups of TYMP and GCNT4 (Log-rank test). (**B, D**) HR values of univariate and multivariate Cox analyses for high and low expression groups of TYMP and GCNT4 (Wald test).
